# COVID-19 Epidemiology during Delta Variant Dominance Period in 45 High-Income Countries, 2020–2021

**DOI:** 10.3201/eid2909.230142

**Published:** 2023-09

**Authors:** Christine J. Atherstone, Sarah Anne J. Guagliardo, Anthony Hawksworth, Kevin O’Laughlin, Kimberly Wong, Michelle L. Sloan, Olga Henao, Carol Y. Rao, Peter D. McElroy, Sarah D. Bennett

**Affiliations:** Centers for Disease Control and Prevention, Atlanta, Georgia, USA

**Keywords:** COVID-19, SARS-CoV-2, coronavirus disease, severe acute respiratory syndrome coronavirus 2, viruses, respiratory infections, zoonoses, epidemiology, COVID-19 testing, morbidity, mortality, primary prevention, vaccination coverage, demographic factors, population density

## Abstract

The SARS-CoV-2 Delta variant, first identified in October 2020, quickly became the dominant variant worldwide. We used publicly available data to explore the relationship between illness and death (peak case rates, death rates, case-fatality rates) and selected predictors (percentage vaccinated, percentage of the population >65 years, population density, testing volume, index of mitigation policies) in 45 high-income countries during the Delta wave using rank-order correlation and ordinal regression. During the Delta-dominant period, most countries reported higher peak case rates (57%) and lower peak case-fatality rates (98%). Higher vaccination coverage was protective against peak case rates (odds ratio 0.95, 95% CI 0.91–0.99) and against peak death rates (odds ratio 0.96, 95% CI 0.91–0.99). Vaccination coverage was vital to preventing infection and death from COVID-19 during the Delta wave. As new variants emerge, public health authorities should encourage the uptake of COVID-19 vaccination and boosters.

The Delta variant of SARS-CoV-2, first identified in India in October 2020 (*1*), became the dominant variant in >130 countries worldwide during June–November 2021 (*2*). Global vaccination coverage during that time remained low; <50% of the world’s population had received >1 dose and <25% had completed a primary vaccination series (*3*). Despite the increased transmissibility of Delta compared with previous variants, countries experienced varying levels of illness and death (*4*).

With each new wave of COVID-19, governments’ responses varied depending on the understanding of SARS-CoV-2, variant characteristics, and societal factors such as healthcare system capacity, vaccination coverage, and the public’s willingness to follow public health mitigation measures. Policies ranged from flattening the curve (i.e., slowing down infection rates to alleviate pressure on healthcare systems) to zero-COVID policies that aimed to completely prevent infections in the community (*5*–*7*).

As the COVID-19 pandemic continues and new variants emerge (*8*), governments are working to identify and implement a combination of effective yet socially and economically acceptable measures. For example, previous works showed the effectiveness of nonpharmaceutical interventions (e.g., remote work, mask-wearing in indoor public spaces) in reducing case rates and death rates (*9*–*11*). In addition, 1 study showed that fewer deaths were reported in countries with earlier and more stringent mitigation policies, such as business closures, restrictions on public gatherings, and stay-at-home orders (*12*). Some countries employing zero-COVID policies experienced rising cases and deaths during the initial Omicron period and beyond (*13*). The benefits and social acceptability of prolonged lockdowns and mass testing policies to prevent new cases and deaths remain unclear (*14*).

We aimed to describe the epidemiology during the pre-Delta and Delta wave periods of the COVID-19 pandemic among high-income countries (HICs) and characterize the relationship between public health policies and epidemiologic burden. Specifically, we assessed the degree to which key measures of illness and death (case rate, death rate, and case-fatality rate) were correlated with vaccination coverage, testing volume, mitigation stringency, population density, and demographics.

## Methods

### Inclusion Criteria

We restricted our analysis to HICs because more accessible testing in those countries likely provides a more accurate assessment of the case and death burdens (*15*,*16*). We included HICs that reported >200 SARS-CoV-2 sequences during the full period of interest (April 1, 2020–November 30, 2021) and >20 Delta sequences in the first month of Delta dominance. The cutoff of 200 sequences was chosen to ensure adequate reflection of variant burden. In August 2021, the World Health Organization (WHO) recommended a minimum of 15 specimens per week (*1*); 200 sequences would provide >3 months’ worth of data. We chose 20 Delta sequences as a minimum to prevent the overrepresentation of a few sequences during weeks that had slower reporting. We defined Delta dominance as the time during which >50% of sequenced samples were Delta.

### Data Sources

We obtained data for this study from 4 publicly available sources. First, we used World Bank country income classifications for 2020 (*17*) to select HICs. This classification is updated annually on July 1 on the basis of the gross national income per capita data from the previous calendar year. Second, information about when the Delta variant became dominant in each country was gleaned from SARS-CoV-2 genomic sequences published in the GISAID (*18*) and GenBank (*19*) databases. Last, we used Our World in Data (OWID) (*15*,*20*) to obtain data on confirmed cases, deaths, percentage of the population fully vaccinated, percentage of the population >65 years of age, population density, SARS-CoV-2 testing volume, and stringency (a composite index of 9 mitigation policies and strategies). OWID compiles data from Johns Hopkins University, World Bank, national government reports, and Oxford University. Data were reported daily (confirmed cases and deaths, stringency index), every weekday (vaccinations), weekly (testing), or annually (population >65 years of age, population density) during the period of interest.

### Data Management

We downloaded sequence data from GISAID and GenBank, aggregated at the country-level, and removed duplicate sequences that were in both databases. Then, for each country, we calculated the weekly proportions of Delta variant sequences during the period of interest, April 1, 2020–November 30, 2021.

To enable easy comparisons between the periods before and after the emergence of the Delta variant, we defined the Delta dominance date as the Monday of the first week in which >50% of sequenced samples were Delta. We defined the pre-Delta period as April 1, 2020, to the Delta dominance date and the Delta dominance period as the Delta dominance date through November 30, 2021. 

### Outcome and Predictor Variables

We used measures of illness and death as outcomes. For the pre-Delta and Delta dominance periods, we calculated the peak 7-day rolling averages for case rates, death rates, and case-fatality rates (CFRs) per million persons. We chose the 7-day peak rolling average to indicate the intensity of the outbreak while also minimizing the effect of bulk data uploads. CFRs used a 14-day lag between daily new cases and daily new deaths (*21*) and was calculated as a ratio of reported deaths to reported cases. Death rates and CFRs offer different types of information about deaths: death rates account for the probability of death in an entire population, whereas CFRs only measure the probability of death among those with the disease. Adherence to case and death reporting might vary by country. To standardize outcomes across countries, we calculated a Delta dominance to pre-Delta ratio (DD:PD) for each outcome. We calculated 3 outcomes of interest as a 7-day rolling average: peak case rate DD:PD, peak death rate DD:PD, and peak CFR DD:PD.

We classified outcome variables into quartiles to enable easier interpretation and statistical evaluation of small datasets. For example, the outcome variable peak case rate DD:PD predictor variables included the percent of the total population fully vaccinated with a primary series at the date of Delta dominance, the percentage of the population >65 years of age, and population density (persons/km^2^). We also included the median 7-day rolling average daily testing volume DD:PD (comparing the period of Delta predominance to the pre-Delta period, as described). Last, we calculated the median stringency index DD:PD. The stringency index, as defined by other scholars (*22*), ranges from 0–100 (100 = strictest) and increases over time if more stringent mitigation policies are implemented or decreases if policies are rescinded (*23*).

### Statistical Analysis

**Figure 1:**
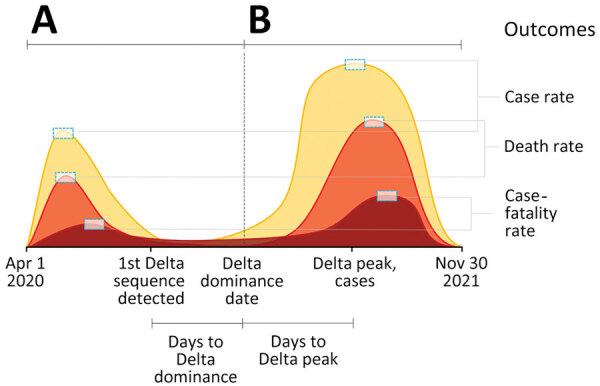
Example of COVID-19 outcomes during pre-Delta variant (A) and Delta dominance (B) periods. Ratio of Delta-dominant period to pre-Delta period is shown for peak case rate, peak death rate, and peak case-fatality rate.

We managed and analyzed data using SPSS Statistics 1.0.0.1406 (IBM Corp., https://www.ibm.com) and RStudio 1.4.1717 (PBC, https://posit.co/blog/rstudio-pbc). To characterize the speed at which Delta supplanted previous variants, we calculated the number of days from the date of the first Delta sequence collection to the date of Delta dominance and from the start of Delta dominance to peak case and death rates (Figure 1). We calculated descriptive statistics (range, mean, median, peak, quartiles) for all measures of illness and death and predictor variables during the pre-Delta and Delta dominance periods.

We used Spearman rank-order correlation to assess the strength and direction of the association between quartile outcomes with predictor variables. For bivariate correlations with p values <0.25, we further assessed relationships between outcomes and predictors in multivariable ordinal regression models. Each of the outcomes were modeled separately. We explored all possible combinations of predictor variables meeting the above criteria and selected the model with the lowest Akaike Information Criterion score as the best model. We evaluated multicollinearity among the predictor variables using a Condition Index cutoff of <15. This activity was reviewed by Centers for Disease Control and Prevention and conducted consistent with applicable federal law and CDC policy (see, e.g., 45 C.F.R. part 46.102(l) (*2*), 21 C.F.R. part 56; 42 U.S.C. §241(d); 5 U.S.C. §552a; 44 U.S.C. §3501 et seq.).

## Results

### Descriptive Analysis

**Figure 2:**
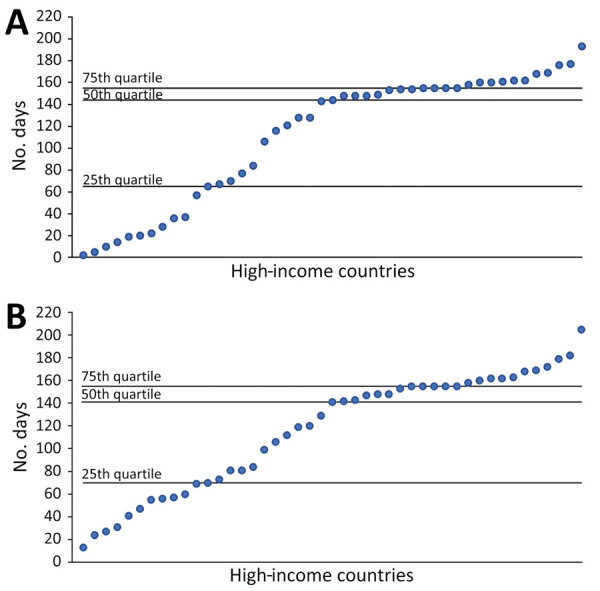
Number of days from SARS-CoV-2 Delta variant dominance to peak case rates (A) and peak death rates (B) in 45 high-income countries, December 2020–November 2021. Each dot represents an individual high-income country. The Delta dominance date for each country was defined as the Monday of the first week in which >50% of sequenced samples in GISAID (https://www.gisaid.org) were Delta.

Among 79 HICs, 45 met the inclusion criteria. The first Delta sequence collection dates ranged from December 28, 2020, to July 26, 2021; more than half (n = 25, 55%) of the countries reported a Delta sequence by the end of March 2021. The median time between the first Delta sequence collected to the start of the Delta dominance period was 77 days (interquartile range [IQR] 49–140). The median time from the start of the Delta dominance period to the peak case rates was 144 (IQR 65–155) days and to the peak death rate was 141 (IQR 70–155) days (Figure 2). Weekly case incidence during the Delta dominance period varied by country and WHO region (Appendix Figure). Average peak case rates ranged from 180 in the WHO Western Pacific region to 1,699 in the African region.

**Figure 3:**
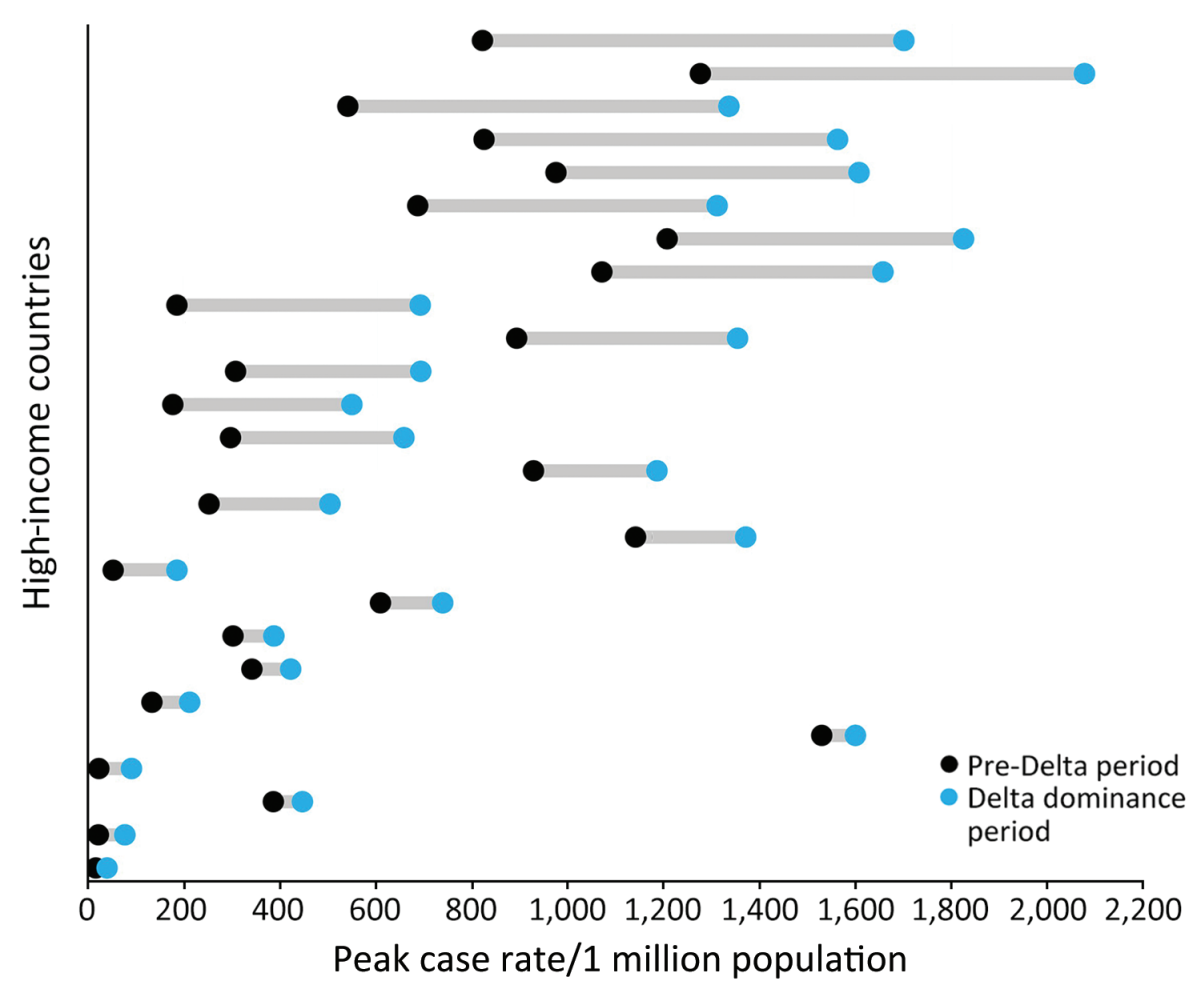
Countries with increased peak COVID-19 case rates during Delta dominance period compared with pre-Delta period in study of COVID-19 epidemiology in 45 high-income countries, December 2020–November 2021. Each data line represents 1 country (n = 26 countries).

**Figure 4:**
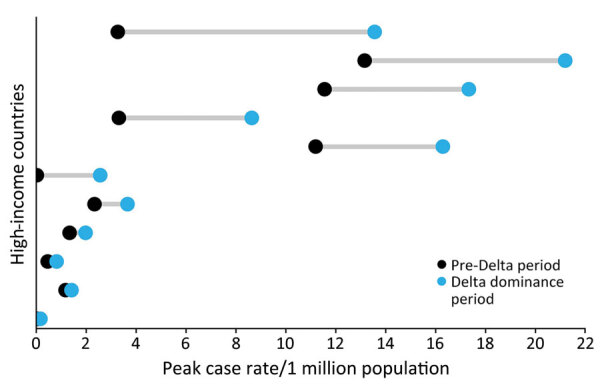
Countries with increased peak COVID-19 death rates during Delta dominance period compared with pre-Delta period in study of COVID-19 epidemiology in 45 high-income countries, December 2020–November 2021. Each data line represents 1 country (n = 11 countries).

Most countries (57%, n = 26) reported higher peak case rates (Figure 3) but lower peak CFRs (98%, n = 44) and peak death rates (75%, n = 34) (Figure 4) during the Delta dominance period than during the pre-Delta period. Ten (22%) countries reported both higher peak case rates and death rates during the Delta dominance period than during the pre-Delta period.

Vaccination coverage with a primary series at the start date of Delta dominance ranged from 1% to 72% in 44 of 45 countries for which data were available (Table 1). The percentage of the population >65 years of age averaged 15.7% (IQR 13.9%–19.5%) in 44 countries for which data were available. Most countries (84%, n = 38) had a lower stringency index during the Delta dominance period than during the pre-Delta period.

**Table 1:** Descriptive statistics for COVID-19 outcomes and predictor variables in Delta dominance period and pre-Delta period for 45 high-income countries, December 2020–November 2021*

Outcome variables: peak rate ratios†	Min	Mean	Median	Peak	IQR
Cases	0.06	1.4	1.2	4.2	0.65–1.91
Deaths	0.05	2.9	0.49	99	0.25–0.93
CFR	0.00	0.21	0.08	2.3	0.02–0.27
Predictor variables					
% Vaccination coverage‡	1.0	32	31	72	24–37
% Population >65 y of age	1.1	16	18	27	14–20
Population density, persons/km^2^	3.2	544	123	7,916	36–237
Median stringency index†	0.5	0.86	0.78	3.5	0.67–0.90
Median daily testing volume†	0.6	2.3	1.7	19.2	1.2–2.4

*CFR, case-fatality rate; IQR, interquartile range. †Delta dominance (DD) period to pre-Delta period ratio. ‡Indicates persons with primary vaccine series at date of Delta predominance.

### Peak Case Rate DD:PD Ratio

Higher vaccination coverage was protective against higher case rates; that is, vaccination resulted in lower peak case rate DD:PD ratios (Spearman rank correlation coefficient [ρ] = −0.36; p = 0.018) (Table 2). The same association was held in the ordinal regression model (odds ratio [OR] 0.95, 95% CI 0.91–0.99; p = 0.01) (Tables 3, 4). There was a positive correlation between median daily testing volume and peak case rate DD:PD ratios (Spearman ρ = 0.30; p = 0.048) (Table 2), although that relationship did not hold in the ordinal regression models (Table 4).

**Table 2:** Spearman rank-order correlations in study of COVID-19 epidemiology in Delta variant dominance period in 45 high-income countries, December 2020–November 2021*

Predictor variables	Peak rate ratio†								
	Cases			Deaths			CFR		
	ρ	p value		ρ	p value		ρ	p value
% Vaccination coverage‡	−0.36	**0.018**		−0.21	0.17		−0.09	0.58
% Population >65 y of age	0.04	0.82		−0.31	**0.039**		−0.48	**0.001**
Population density, persons/km^2^	−0.05	0.75		−0.12	0.45		0.05	0.76
Median stringency index†	0.04	0.78		0.07	0.63		−0.14	0.35
Median daily testing volume†	0.30	**0.048**		0.24	0.12		0.18	0.24

*Bold indicates statistical significance (p<0.05). CFR, case-fatality rate; ρ, Spearman rank correlation coefficient. †Delta dominance period to pre-Delta period ratio. ‡Indicates persons with primary series at date of Delta predominance.

**Table 3:** Ordinal regression results in study of COVID-19 epidemiology in Delta variant dominance period in 45 high-income countries, December 2020–November 2021*

Model	Peak rate ratio outcome†	Predictor variable	OR (95% CI)	p value
1	Cases	% Vaccination coverage‡	0.95 (0.91–0.99)	**0.014**
2	Deaths	% Vaccination coverage‡	0.96 (0.92–0.99)§	**0.025**
		% Population >65 y of age	0.88 (0.78–0.98)§	**0.021**
3	CFR	% Population >65 y of age	0.89 (0.80–0.98)	**0.013**

*Bold indicates statistical significance (p<0.05). CFR, case-fatality rate; OR, odds ratio. †Delta dominance (DD) period: pre-Delta (PD) period ratio. ‡Indicates persons with primary series at date of Delta predominance. §Values shown are adjusted OR (95% CI).

**Table 4:** Heat map of bivariate analyses and ordinal regression model results in study of COVID-19 epidemiology in Delta variant dominance period in 45 high-income countries, December 2020–November 2021*

	Peak rate ratio†						
	Bivariate analysis				Ordinal regression results		
Predictor variables	Cases	Deaths	CFR		Cases	Deaths	CFR
% Vaccination coverage‡†							
% population >65 y of age							
Population density, persons/km^2^							
Median stringency index†							
Median daily testing volume†							

*Blue shading indicates negative (protective) association, yellow shading indicates positive association, and no shading indicates no significant association. CFR, case-fatality rate. †Delta dominance (DD) period: pre-Delta (PD) period ratio.  ‡Indicates persons with primary series at date of Delta predominance.

### Peak Death Rate DD:PD Ratio

A higher percentage of the population being persons >65 years of age was protective against death rates (peak death rate DD:PD ratios), both in bivariate comparisons (Spearman ρ = −0.31; p = 0.039) (Table 2) and in the regression models (OR 0.88, 95% CI 0.78–0.98; p = 0.02). In addition, higher vaccination coverage was associated with lower peak death rate DD:PD ratios (OR 0.96, 95% CI 0.92–0.99; p = 0.03) in the regression models.

### Peak CFR DD:PD Ratio

A higher percentage of the population being persons >65 years of age was associated with lower CFRs (lower peak CFR DD:PD ratios, Spearman ρ = −0.48; p = 0.001) (Table 2). This association held in the ordinal regression model (OR 0.89, 95% CI 0.80–0.98; p = 0.01).

## Discussion

Most HICs had higher case rates but lower death rates and CFRs during the Delta dominance period than in their pre-Delta periods. Achieving higher vaccination coverage appeared to be the main public health measure associated with a decrease in the intensity (peak) of COVID-19 cases and deaths. Each quartile increase in vaccination coverage resulted in a 5% reduction in peak case rates and a 4% reduction in peak death rates. Countries with a larger percentage of persons >65 years of age had lower death rates and CFRs during the Delta dominance period, likely because of focused early vaccination activities in this age group and case management protocols prioritizing older populations for close observation and hospital admission (*24*,*25*).

Many HICs recorded increased case rates during the Delta dominance period, but those increases did not often result in higher death rates than for the pre-Delta period. Previous studies reported mixed results on death rates during the period of Delta variant prevalence; 2 studies reported no differences in mortality rates (*26*) or in-hospital deaths (*27*), and 3 studies indicated an increase in mortality rates (*28*; A. Kumar et. Al, unpub. Data, https://www.medrxiv.org/content/10.1101/2021.09.23.21263948v1) and in-hospital deaths (R.S. Khedar et al., unpub. Data, https://www.medrxiv.org/content/10.1101/2021.09.03.21263091v1). The lower death rates and CFRs among HICs in this study may be the result of advances in therapeutics, improvements in case management protocols, increased natural immunity from previous infection, and the availability of vaccines, rather than decreased virulence of the Delta variant virus (*29*–*31*). Among global studies from HICs, the early prioritization of vaccination in the elderly and achievement of high coverage appeared to protect older adults against death during the Delta dominance period. Studies have shown that, during the Delta wave, vaccines were less effective against infection (*32**,**33*; R.S. Barlow et al., unpub. data, https://www.medrxiv.org/content/10.1101/2021.08.30.21262446v1) but highly protective against symptomatic disease (*29*,*33*), hospitalization (*34*), and death (*33*). This lack of protection against infection among vaccinated persons, combined with the increased transmissibility of the Delta variant, likely contributed to an overall higher case burden during the Delta predominance period in HICs. Nevertheless, despite Delta’s high transmissibility, peak case and death rates occurred in many countries >4 months after the date of Delta’s dominance, which would have allowed time for strategies such as vaccination to be implemented and have an effect (*35*).

The first limitation of this study is that we used publicly available datasets, which might be affected by fluctuations in reporting, including reporting of at-home testing. Death rates might not be a robust indicator of severity in HICs. Hospitalization data might have provided another measure of illness severity, but OWID hospitalization data were available for <50% of countries included in the study and had many reporting gaps. The goal of this study was to identify population-level associations between COVID-19 illness and death and disease control metrics; a priori testing of more specific hypotheses might be addressed in other assessments using different study designs (e.g., a community-wide, cluster-randomized trial that evaluated masking in Bangladesh [*36*]). This analysis was ecologic in nature, and therefore population-level associations might not apply at the individual level. We also focused on the outcomes of peak cases, deaths, and CFRs and therefore did not capture the total burden of cases and deaths. Finally, the variety of COVID-19 vaccines available and changing recommendations on the number of doses for maximal effectiveness make it difficult to generalize the findings associated with vaccination coverage to all COVID-19 variants and coverage effectiveness over time.

In conclusion, this characterization of epidemiologic outcomes in high-income countries during the Delta dominance period shows that many countries reported higher case rates but lower death and case fatality rates compared to the pre-Delta period; higher vaccination coverage and completion of a primary vaccination series were associated with lower case and death rates; and >4 months elapsed between Delta introduction and peaks in case rates and death rates, which might have allowed time for mitigation strategies such as vaccination to be implemented and have an impact. These findings might be useful in informing public health authorities of the importance of achieving high vaccination coverage as the pandemic continues to evolve. The ability to continue implementing measures to combat COVID-19 might be limited by authorities’ willingness to implement stronger measures and the willingness of the public to comply. However, across multiple HICs, our findings consistently indicate that higher vaccination coverage can result in fewer cases and deaths.

Appendix Additional information about COVID-19 epidemiology during Delta variant dominance period in 45 high-income countries, 2020–2021.
