# Supplementary material for: COVID-19 Epidemiology during Delta Variant Dominance Period in 45 High-Income Countries, 2020–2021
Source: Emerg Infect Dis. 2023 Sep;29(9):1757–64. doi: 10.3201/eid2909.230142 (PMC10461680; doi:10.3201/eid2909.230142)
Supplement: Appendix — Additional information about COVID-19 epidemiology during Delta variant dominance period in 45 high-income countries, 2020–2021. [file 23-0142-Techapp-s1.pdf]

Article DOI: https://doi.org/10.3201/eid2909.230142

EID cannot ensure accessibility for supplementary materials supplied by authors. Readers who have difficulty accessing supplementary content should contact the authors for assistance.

COVID-19 Epidemiology during Delta Variant Dominance Period in 45 High-Income Countries, 2020–2021

Appendix

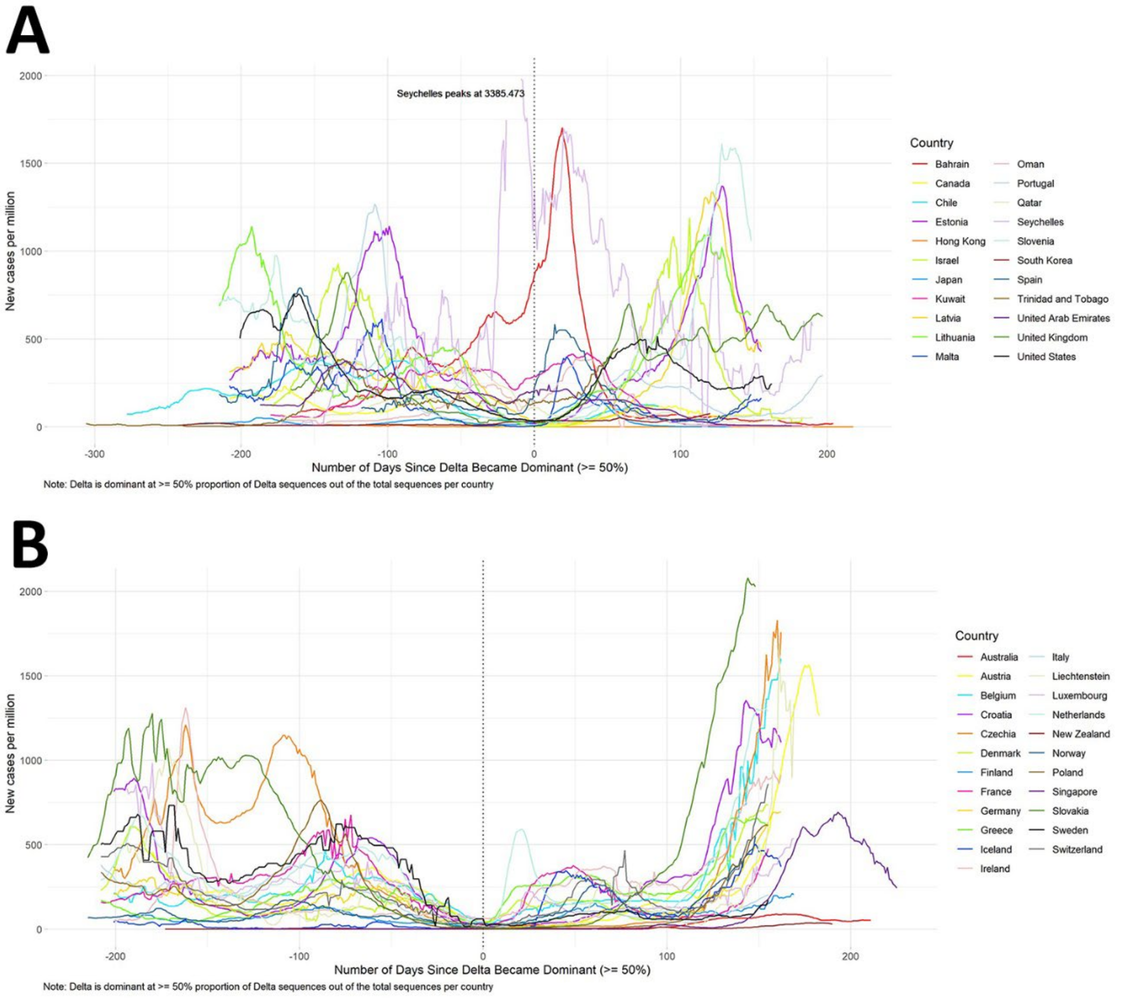

Appendix Figure. A) Weekly new cases by days since Delta dominance* (22 countries with earliest peak case dates). *The Delta dominance date for each country was defined as the first week in which at least

50% of sequenced samples reported in GISAID were Delta. Countries by World Health Organization region: AFRO, Seychelles; AMRO, Canada, Chile, Trinidad and Tobago, United States; EMRO, Bahrain, Kuwait, Oman, Qatar, United Arab Emirates; EURO: Estonia, Israel, Latvia, Lithuania, Malta, Portugal, Slovenia, Spain, United Kingdom; WPRO, Hong Kong, Japan, South Korea. B) Weekly New Cases by Days Since Delta Dominance* (23 countries with latest peak case dates). *The Delta dominance date for each country was defined as the first week in which at least 50% of sequenced samples reported in GISAID were Delta. Countries by World Health Organization region: EURO, Austria, Belgium, Croatia, Czechia, Denmark, Finland, France, Germany, Greece, Iceland, Ireland, Italy, Liechtenstein, Luxembourg, Netherlands, Norway, Poland, Slovakia, Sweden, Switzerland; WPRO, Australia, New Zealand, Singapore.
